# Sustainable Nipa Palm (*Nypa fruticans* Wurmb.) Product Utilization in Thailand

**DOI:** 10.1155/2020/3856203

**Published:** 2020-09-25

**Authors:** Onanong Cheablam, Boontaree Chanklap

**Affiliations:** School of Management, Walailak University, Nakhon Si Thammarat 80160, Thailand

## Abstract

Nipa palms, a plant species in mangrove forest, are valuable and beneficial for the local community's economy as well as the conservation in the southern region of Thailand. This study aimed to investigate the use of nipa palms in Khanap Nak and focused on the type of products made from nipa palms and the yield of this plant through focus group discussion and interview with the farmers maintaining nipa palm forest. The results suggested that nipa palms in Khanap Nak can yield for 5–100 years. Apart from the benefits to the community in terms of sustainability, as it prevents erosion, it provides sources for the production of food (molasses, granulated sugar, syrup, and vinegar from sap) and raw materials (roofing material and cigarette paper from leaves and stem), which can generate income to community members. It was found that most of the communities earn 90–130 USD/day from palm sugar production. The quantity of nipa palm products varies in each season, resulting in different prices. In this regard, they can produce high quantity of products made from sap from January to March. However, the production of different types of sugar requires local knowledge and wisdom to ensure good quality. Nipa palm production is the sustainable way to utilize mangrove forest resources, leading to effective conservation and good life quality. Regarding problems and difficulties in farming, it was found that natural disaster is a major threat, such as drought, excessive amount of salt or freshwater in certain periods, and insect pests.

## 1. Introduction

Thailand is located in the tropics, which has a diversity of mangrove forest [1]. Various species of plants in mangrove forest can be potentially processed in many different ways to support the local livelihood, such as food, fuel, medicine, and construction materials [2, 3]. Considered one of the oldest living palms [4], the nipa palm (*Nypa fruticans* Wurmb.) is the only species able to adapt to mangrove forest. This trunkless palm dominates wetlands commonly flooded by brackish water [5–7]. This mangrove palm is harvested to produce various products depending on tradition and lifestyle, including sugar syrup from its floral and fruit stalk exudate; thatching materials, cigarette paper, and construction materials from its leaves; as well as food and dessert from its fruits [3].

Located in Pak Phanang Basin, Khanap Nak is the largest source of nipa palms in Thailand [1]. Nipa palms' sap locally produced in the Pak Phanang Basin is rich in nutrition and chemical components, such as sucrose, glucose, and fructose [8, 9].

Sustainable utilization of nipa palms can make environmental, social, and economical impacts [10]. The sap is used to produce many products for consumption, such as fresh juice, syrup, alcohol, molasses, and traditional vinegar, which can be sold to generate income to the community, resulting in very promising economic potential. In addition, many local communities inherit traditional techniques for generations to utilize nipa palms that remain a part of the community's livelihood [1]. Currently, a few government agencies support and promote the development of abandoned shrimp farms for nipa palm cultivation and the utilization of vacant spaces to increase the abundance of mangrove forest. Consumable products from nipa palms in Pak Phanang Basin are obtained through traditional extraction techniques, and some resource development strategies have been implemented in this area.

Centuries ago, coastal communities in Pak Phanang Basin have traditionally been utilizing nipa palms, but the empirical evidence of production and consumption of the processed products from nipa palms is still limited. Similarly, the value of nipa palms on environmental, social, and economical aspects is the contribution to sustainable utilization of forest products. In connection with this, the present study aimed to identify a variety of nipa palms' sap products utilized by local communities in Pak Phanang Basin. It was expected to contribute to the literature regarding the role of nipa palms in these communities, income generation, and its environmental value.

## 2. Study Site

**Figure 1:**
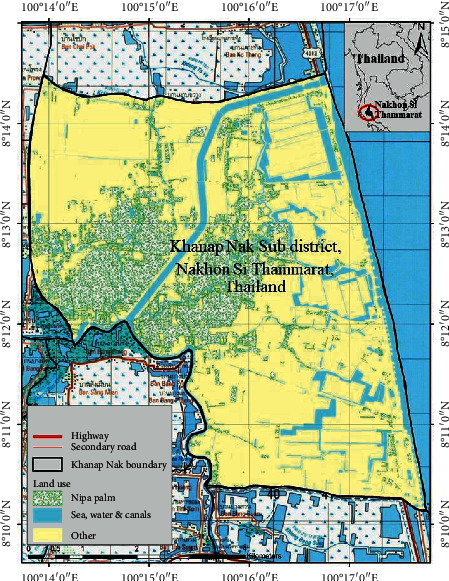
Khanap Nak, Nakhon Si Thammarat, Thailand.

This research was conducted in Khanap Nak, located in Pak Phanang Basin, Nakhon Si Thammarat, Thailand, which is the largest source of nipa palms in Thailand [1, 9]. The area of study is located at 8°10′8″N to 8°14′30″N and 100°13′48″E to 100°17′46″E (Figure 1). Topographically, the site is a swampy area with a moderate tidal influence [1, 11].

## 3. Materials and Methods

Data were obtained from two sources: primary and secondary. The primary data were collected from January to October 2019, while the secondary data were gathered or collected from various sources, such as documents issued by the Department of Marine and Coastal Resources, journal articles, official records, and project reports. The values, lifestyles, and importance attached to nipa palms as well as its role in income generation were investigated. The process involved the following methods:

1. *Key Informant Interview*. The selected key informants for this research were related to nipa palms, including the community representatives who were directly and indirectly involved in the production and distribution of nipa palm products. They were selected through snowball sampling based on the characteristic suitability of the subjects and the requirements to obtain complete, accurate, and consistent data. There were a total of 12 informants participating.
2. *Brainstorming and Focus Group Discussion*. This allowed people with nipa palm plantation to participate in providing data regarding values, products, and lifestyles related to nipa palms.
3. *Interview with 100 Households (of 389) of Nipa Palm Producers from 10 Villages*. The sample size of this study is more than 25% of the population for the small population [12]. The structured interview focused on the categories, types, volume, and preparedness of the community to produce nipa palm products. The respondents for the study were sampled by using a simple purposive sampling technique to select the well knowledgeable and experienced people with nipa palm–related occupation who were willing to provide the nipa palm information.
4. *Survey and Direct Observations of Nipa Palm Forest and Farms in Khanap Nak.* The productivity of each farm of people was surveyed to determine the yields.

**Figure 2:**
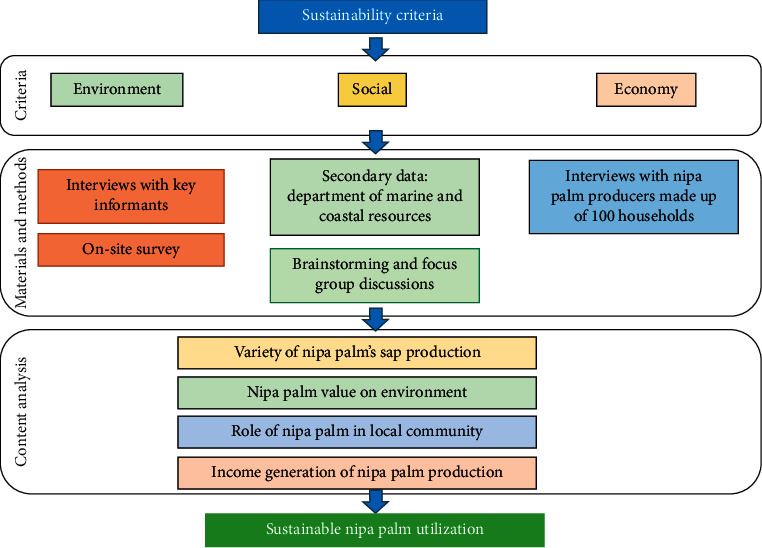
Methodological framework.

This study specified the sustainability criteria to assess the opportunities of nipa palm product utilization from environmental, social, and economical sustainability aspects (Figure 2). The obtained data were analyzed by a qualitative descriptive analysis focusing on both measuring the volume and income and interpreting the types and process of nipa palm products. All results were illustrated using charts, pictures, and tables.

## 4. Results and Discussion

### 4.1. Sustainable Utilization of Nipa Palm

**Figure 3:**
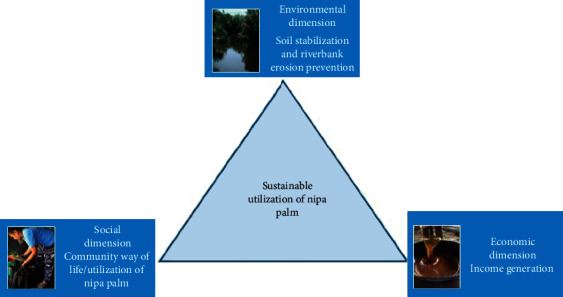
Sustainable utilization of nipa palms.

Khanap Nak has an area of approximately 38.8 square kilometers with 10 villages and a total population of 5,295 people. The community is a coastal community with a large amount of nipa palms. Therefore, the community's lifestyle is linked with nipa palms and people rely on making nipa palm products for primary and supplementary income. According to the findings of this study, the community is a home to 389 households relying on nipa palms for living. A survey indicated the palm forest area of 5.76 square kilometers in this community. The results from the farmer interview revealed the age of palm trees in this area is from 5 to 100 years. In addition, it was found from the interview and observation that nipa palms are crucial for the community in environmental, social, and economical aspects as presented in Figure 3.

As pointed out earlier, nipa palms are a valuable resource in coastal areas of Thailand. Local communities rely on this plant for both environmental and economic benefits [1]. Particularly in the southern region, nipa palms' sap can be processed to dessert, syrup, and vinegar [9, 13]. Many reports such as the one by Hossain and Islam in Bangladesh showed that the sap is useful for making sugar. Nipa palms are also utilized for roof thatching and wall partitioning and as food and medicinal purposes [14]. In terms of conservation, nipa palms have the conservation value in the area where they are grown, including conserving local community lifestyles and natural resources [3, 14]. The value and importance of nipa palms are discussed in detail in the following.

### 4.2. Environmental Dimension: Soil Stabilization and Riverbank Erosion Prevention

**Figure 4:**
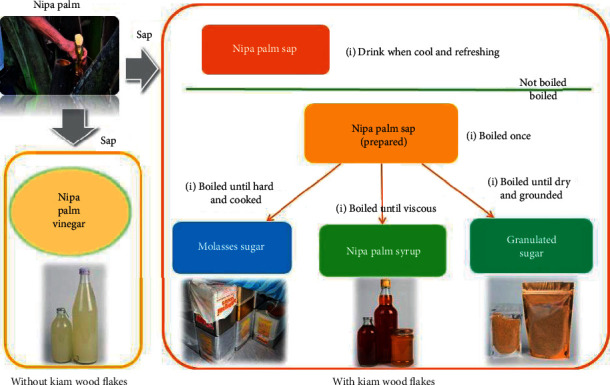
Sap processing into various products using traditional extraction techniques.

The knowledge about nipa palm cultivation is acquired through inheritance and has been passed on from generation to generation by the villagers of Khanap Nak. Since ancient time, the villagers have followed traditional methods for cultivating nipa palms and producing nipa palm sugar. The cultivation goes through a series of methods and processes and involves various equipment. The extraction also involves traditional techniques as shown in Figure 4. In addition to being a source of raw materials, nipa palm forest is an excellent line of protection against erosion and collapse of riverbanks. The survey showed that nipa palm grows well in saline soil or mud next to rivers or canals flooded at all times or in lowland areas with depleted soil. In some seasons, nipa palms can grow in highland area with proper flooding. However, nipa palms growing next to riverbanks or mangroves are capable of spreading their roots to cover a wider area in the soil. Thus, the nipa palm promotes biodiversity, prevents coastal erosion, and provides a habitat for aquatic animals and food for members of the community [3].

### 4.3. Social Dimension: Community Way of Life/Utilization of Nipa Palm

Every part of a nipa palm can be used for a variety of purposes. As cooking ingredients, nipa palms were found to be beneficial to people in the communities where the study was conducted. In Khanap Nak, young flowers were used to make food, especially by boiling and then dipping in chili paste. Similarly, fruits aged four months were pickled and cooked as a vegetable in curry. Fruits aged 5–8 months were used to make dessert, while sap from nipa palm flowers were used to make sugar. In addition, the sap can be used to make nipa palm sugar, powdered sugar, molasses, and vinegar. Phetrit et al. [8] reported that nipa palm syrup has a potential source of nutrients and bioactive components as it contains 3 major types of sugar (96.53% dry weight), including sucrose (78.29% dry weight), glucose (9.83% dry weight), and fructose (6.78% dry weight). Additionally, this syrup has minerals, polyphenols, mallard reaction products (MRPs), caramelization products (CPs), and other bioactive components.

In an interview, a farmer supporting the health value of nipa palms said the following.

“*I have been eating nipa palm sugar since I was little. Also, my ancestors regularly consumed nipa palm sugar and no one has had diabetes, everyone is healthy*.” (Nipa palm farmer from Village No. 5, interviewed on April 8, 2019).

The preparation processes for each type of nipa palm syrup are summarized in Figure 4. The sugar-making process begins by collecting sap. The flowers and fruits are removed, and then the inflorescence is cut to produce sap [1, 15]. Nipa palms' sap without kiam wood flakes added is fermented for 10–15 days for vinegar production. Kiam wood (*Cotylelobium lanceolatum* Craib), sliced into small flakes, is added into the bamboo cylinder in the extraction process. It contains polyphenols with acerbic taste, which act as antimicrobial and antioxidative agents [1, 16, 17]. However, if kiam wood flakes are added into the cylinder while collecting the sap, the sap can be used to make several other types of sweetened beverages. The sap can be consumed raw. Once boiled, it can be consumed as a beverage. To make syrup, the sap is boiled until viscous and left to cool in a bottle. In addition, the cooled syrup can be dried and then grinded to produce powdered syrup. Regarding molasses, the sap is boiled until viscous and cooled down by stirring with a wooden paddle until thickened. It is then packed in buckets.

Each type of sap requires different preparation methods and equipment based on community knowledge, which has been transferred from generation to generation. Each step requires experience and expertise, such as the insufficient addition of kiam wood flakes can cause sap to rot, while the excessive addition can cause too much bitter taste and ultimately affect the taste of the processed sap. Nipa palms' sap products can then be distributed and consumed in the household.

In addition to using nipa palms for consumption, it was revealed that the residents in Khanap Nak use nipa palms to make various products; for example, water containers made of leaves, ropes for thatch sewing, and potholders made of soft branches. In some locations, fresh nipa palm tops aged two months are used to roll cigarettes, while old leaves and branches are whittled to make sticks of 1–1.20 meters length to hold thatches while sewing. The old leaves can be also woven into hats.

### 4.4. Economic Dimension: Income Sources for Local Community

Apart from consumption benefits and conservation value, nipa palms provide crucial benefits and values in improving the quality of life of local communities through generating income for many households. It was evident from the interview conducted with community members in Khanap Nak that nipa palm farming is a supplemental occupation for some households and a primary one for many households all year round. According to the interview, the households cultivating nipa palms as well as those making various products from the crop derived income directly from the venture. For instance, molasses can be sold at a price of 900–1,400 THB (30–46.7 USD) per bucket (25 kilograms), depending on the season, while granulated sugar at a price of 120–140 THB (30 USD) per kilogram and bottled syrup at a price of 60 THB (2 USD) per bottle. However, the income generated from nipa palm sugar is dependent on the quality of produced sugar influenced by factors, such as water quality, rainfall, and weeds.

### 4.5. Nipa Palm Products: Wisdom Products and Income Sources

**Figure 5:**
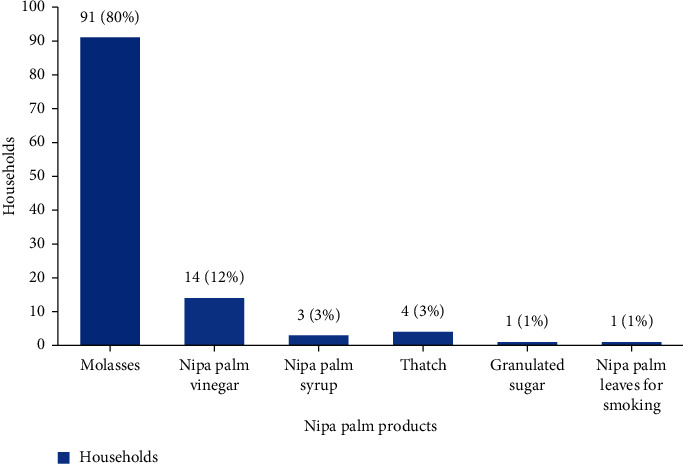
Nipa palm products and production volume in Khanap Nak and Pak Phanang.

In terms of utilization, different products were found to be made from and of nipa palms in all 10 villages of Khanap Nak (Figure 5). Nipa palm sap contains high concentrations of minerals and is rich in sugar (Phetrit et al. [8]).

The results from the survey and interview with 100 households engaged in nipa palm production showed that the dominant products from nipa palms were molasses (91 households or 80% of farmers), followed by vinegar at 12%, syrup and thatch at 3% each, and others, such as granulated sugar and cigarette paper, at 1%. Similarly, nipa palm molasses is one of the most important products invaluable for the economy of Bangladesh [14].

**Figure 6:**
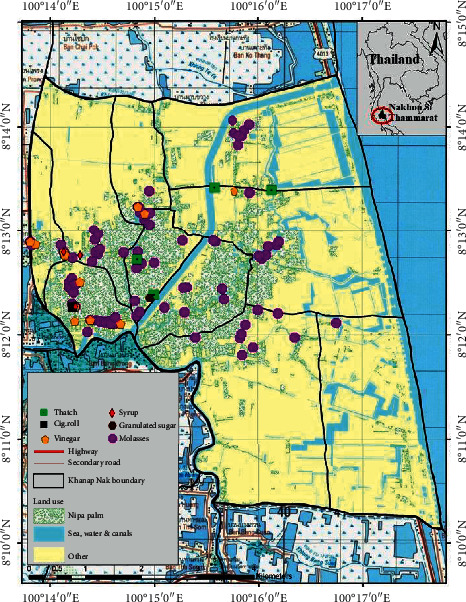
Dispersion of products from nipa palm forest resources in Khanap Nak, Pak Phanang.

According to Figure 5, most production from nipa palms was in the form of molasses. The farmers dispersed the production throughout the subdistrict, particularly in Village Nos. 2, 4, 5, and 6. Regarding vinegar, the production was found largely in Village Nos. 1, 2, 4, and 7 with none found in other areas of the subdistrict. Furthermore, the syrup or liquid sugar production occurred in Village Nos. 1, 2, and 3 with only one household found to be involved in production. The thatch production was found in Village Nos. 5, 7, and 10, and the granulated sugar production was found in only one household in Village No. 5. Finally, the cigarette paper production was found in only Village No. 2 as shown in Figure 6. The aforementioned production is undertaken in the form of a community enterprise where community members gather to produce various products for distribution.

## 5. Products from Nipa Palms' Sap

The nipa palms' sap is a major ingredient in many products. As indicated, the farmers and community members use the traditional extraction techniques to produce these products as a way to generate income to support their living. Four main products obtained from the sap are discussed in detail as follows.

### 5.1. Molasses

**Figure 7:**
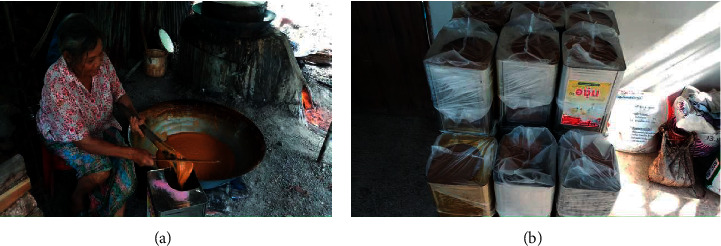
Farmer and molasses production.

The molasses production is the most popular product in Nakhon Si Thammarat. The interview with nipa palm farmers and survey results indicated that molasses is the main product for alcohol production (Figure 7). This product can generate 33,120 THB (1,104 USD) per month to the community, and the farmers can produce molasses at an average of 0.96 buckets per day, while some can produce a maximum of 3 buckets per day. The molasses production requires approximately 176.26 stalks per household, considered high rate. The interview indicated that 5–6 liters of sap is required to produce 1 kilogram of molasses. However, Hossain and Islam [14] reported that in Bangladesh, 1 kilogram of molasses was produced from 7–8 liters of sap. Furthermore, in the area of nipa palm farms, the molasses production required an average of 2 persons. It was found that the experience of the households with molasses production was at an average of 26 years.

It was observed that most of the molasses producers are elderly local people with an average age of 58 years, while nipa palm farmers had an average age of 85 years with extensive experience in farming on their own land and/or leased land. Therefore, this area has adequate labor force to engage on nipa palm farming. According to the interview, the nipa palm farmers 90.11% chose to make molasses because it is easy to sell the products to middlemen who buy directly from the farmers. In addition, the farmers indicated that molasses is in demand and can be sold throughout the year even though the price may insignificantly vary throughout the year. A farmer in Village No. 2 indicated the following in an interview.

“*Molasses is easy to make, uncomplicated and, most importantly, it can be kept for years. I sometimes store 10–20 buckets before calling the merchant to come and pick it up at my house. I get a lump sum for it.*” (Nipa palm farmer from Village No. 2, interviewed on April 7, 2019).

### 5.2. Nipa Palm Syrup

Nipa palm syrup is a popular product for consumption in Khanap Nak and other areas and for purchase by tourists visiting the region. However, few producers were found in this study. According to a survey, there were only 3.

In an interview with a farmer in Village No.5, he spoke about nipa palm syrup production:

“*Only few people make nipa palm syrup. Most of them don't. They've all gone on to make* molasses *because it's difficult and time-consuming to make nipa palm syrup. Most importantly, the cylinder and sap need to be kept clean.*” (Nipa palm farmer from Village No. 5, interviewed on May 5, 2019).

It was found that Khanap Nak can produce nipa palm syrup at an average of 28.33 liters per day and a maximum of 60 liters per day. The spadices are currently required at an average of 126.67 spadices per household.

In terms of labor availability, the community has 2.33 workers for production per household with an average age of 56 years and 17 years of experience in making nipa palm syrup.

Regarding the choice of production, it was found that the farmers chose to produce nipa palm syrup due to market availability and continual purchase. The products can be sold in wholesale and retail sales at all time, resulting in continual production. However, the syrup production process is very detailed and the sap's purity or cleanliness needs to be maintained throughout the production process.

### 5.3. Granulated Sugar/Sugar Flakes

**Figure 8:**
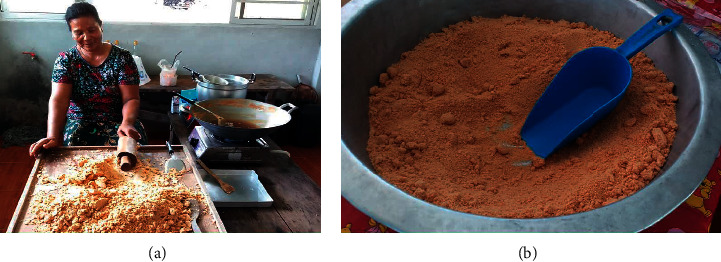
Granulated sugar in Khanap Nak.

The sugar flake production in Khanap Nak was found to be low. A survey of the communities showed that Village No. 5 has only 1 producer with an average production of 11.5 kilograms per day. 150 spadices are required for the production per household, and 5 workers engaged in production. As highlighted in Figure 8, the powdered sugar/sugar flake production requires a large number of people due to complicated process and the need of high level of experience. In Village No. 5, the farmers has 30 years of experience in making sugar flakes, implying that she has the capability to make quality sugar flakes from nipa palm sap.

Regarding the choice of production, the market demand for the product is the key factor. Furthermore, the produced sugar flakes can be easily sold in wholesale and retail sales. Despite the process requiring detailed procedures and patience, the diligence and need to sell different products from elsewhere prompt the farmers to produce sugar flakes for distribution. Thus, the sugar flake production was merged to the community's enterprises.

As Hossain and Islam [14] reported the nipa palm utilization in Bangladesh and Kusmana [3] presented the mangrove utilization in Indonesia, the granulated sugar made from nipa palm sap was not mentioned. Therefore, the granulated sugar production in Khanap Nak will increase the knowledge about the production pattern and value of nipa palm.

### 5.4. Nipa Palm Vinegar

East Asia is commonly known for nipa palm vinegar consumption [18]. Nipa palm vinegar is produced by traditional methods and used as a food ingredient and preservative agent [19]. The production of nipa palm vinegar makes up 12% of the total production in Khanap Nak, and the farmers can produce 38.21 liters per day. The interview results showed that the volume of produced vinegar can be as high as 200 liters per day in Village No. 2. Moreover, it was found that the nipa palm farmers cut an average of 130 spadices per household with the largest number of spadices owned by farmers at 400. Regarding the labor for vinegar production, the area has an average of 1.8 workers per household. The farmers producing nipa palm vinegar have an average age of 62 years and nipa palm farming experience at an average of 36 years.

It was also revealed during the interview and survey that nipa palm vinegar is a healthy product and can be used to cook many types of local food. The nutrition and chemical components, such as sucrose, glucose, fructose, vitamins, and antioxidant activities, were found in nipa palm sap [8, 9, 20]. Many local communities in Malaysia use nipa palm vinegar as medicine to treat diabetes [21].

## 6. Other Products from Nipa Palm

### 6.1. Nipa Palm Thatch

Nipa palm thatch is another important product from nipa palm for making roofs and walls of dwellings [6, 22, 23]. According to the survey on nipa palm thatch production in Khanap Nak, 4 farmers have nipa palm farms with thatch production at an average of 60 rows per day. Some of the farmers can produce as much as 100 rows per day. The labor availability for thatch production stood at an average of 1.5 workers, most of whom are aged 60 years with 21.33 years of experience in making nipa palm thatch.

Regarding the choice of production, 50% of the farmers were found to use materials, such as nipa palm leaves. In dry season, the branches are trimmed and the trimmed leaves are used to produce thatch for sale and use in the community. In addition, the farmers indicated that thatch is easy to sell as the demand was high. After the production, they sell the remaining trimmed leaves to the merchants in the community. More importantly, they can benefit from low production costs due to the availability of nipa palm leaves in the area. Furthermore, ropes from khla trees grown in Khanap Nak are used in the production process. This study shows that leaves are less utilized than the sap. This finding is in contrast with the situation in Bohol, Philippines, where nipa palms are more utilized for thatch production than any other purposes [5].

### 6.2. Nipa Palm Cigarette Paper

In the past, people smoked cigarettes using nipa palm leaflets for cigarette paper [6, 24], which remain popular among the communities in rural areas. The cigarette paper production from nipa palm leaves used to be only for household and community consumption. In the area of Khanap Nak, only 1 farmer in Village No. 2, an elderly person, was found to produce nipa palm cigarette paper based on the survey, at an average of 100 bags per day.

In addition to product distribution, it was evident from the findings and discussion that nipa palm products have benefits and values in terms of improving the community's quality of life and generating income. The interview with the community members showed that the households engaging in nipa palm cultivation have the maximum daily income from molasses at 2,700–3,000 THB and the farmers can profit 1,200–2,000 THB per day maximum (Table 1).

Despite the production of various products from nipa palms, it is crucial to point out that nipa palm producers still face 4 main problems (Table 2). Most of the farmers involved in the production are old and easily exhausted. Therefore, there should be a system in place to ensure that their experiences are inherited to the younger generation. In terms of funding, it was found that most of the farmers encounter few challenges as most of them rely on natural resources and organic farming without pesticides and herbicides, resulting in chemical-free products and low production cost. For equipment, they indicated that it is still sufficient because they rely on locally made tools, which is inexpensive, except for kiam wood purchased from other sources. The main problem for nipa palm cultivation is natural disasters, including changes in the water salinity level and drought. The growth properties of nipa palm fruit stalk depend on the status of water and the yield is influenced by the treatment [25]. The study showed that nipa palm grows well in brackish water [13]. Therefore, when there is too much seawater or freshwater, it can affect the quality of sap or sugar production. Matsui et al. [13] also reported that as the amount of Na^+^ in soil and the nipa palm growth control the sap production, it can be improved by both increasing the soil organic matter content and decreasing brackish water. In addition, the average maximum temperature for nipa palm is 32–35°C, and the optimal climate is subhumid to humid [14]. The result of the study showed that the drought in 2019 affected the flowering of nipa palm in many areas.

In an interview with a farmer in Village No. 1, he spoke the following.

*“This year is hot …no rain in the nipa palm for 8 months, so the flower inflorescence of nipa palm cannot grow and no sap for a long time. ”* (Nipa palm farmer from Village No. 1, interviewed on April 14, 2019).

Sampson et al. [26] admitted that livelihood activities amidst climate variability are threatened, especially those that are climatic-dependent like smallholder farming. Therefore, when the temperature is high, the productivity of sap of nipa palms is affected.

### 6.3. Nipa Palm Forest Management

Nipa palm forest is a source of food and raw materials for household instrument production and income generation for many local communities. The results of the study indicated the main products from nipa palm sap are sugar, vinegar, and alcohol, similar to the production in Bangladesh [27].

Therefore, the communities in the study area have maintained the nipa palm forest to prevent soil erosion and increased the forest size. The community members participate in the nipa palm forest management with oversight and support for sustainable utilization from the government agencies and implement the following management strategies:

1. Allow the community participation in maintaining nipa palm forest through management, planning, and utilization. Many communities have inherited the maintenance and utilization methods for nipa palm forest from generation to generation for a long period of time. Therefore, they adapt the principles of the sustainability management while benefiting from the resources provided by the forest.
2. Maintain the good price for production and distribution of nipa palm products while supporting markets for the community to continually generate income.
3. Develop nipa palm products to meet required standards with longer shelf life to increase product value.

## 7. Conclusion

Khanap Nak is an area with high nipa palm cultivation and has utilized nipa palm to produce a variety of products for a long time. In particular, nipa palm syrup production is part of local knowledge and cultural heritage of the area. The leaves, branches, and flowers of nipa palms can be used for cooking, while the sap from the spadix can be used to make sugar and vinegar. Therefore, nipa palms and mangrove forest have diverse benefits and values in terms of environment, utilization, and income generation. The communities cultivating nipa palms have primary and supplemental occupations related to nipa palms. In conclusion, Khanap Nak is a model area for sustainable nipa palm utilization. Given the tropical area is optimal for nipa palm growth, the sustainable utilization would benefit the forest and improve life quality of people in the community.

## Figures and Tables

**Table 1:** Products from nipa palm in Khanap Nak.

Item	Mean	Max.	Min.	Max. income (baht/day)	Min. income (baht/day)
*Molasses*					
Daily production (buckets/day)	0.96	3	0.5	2,700–3,900 (90–130 USD/day)	450–650 (15–21.7 USD/day)
No. of stalk cut (stalk/household)	176.26	400	50		

*Syrup*					
Daily production (liters/day)	28.33	60	5	6,000 (200 USD/day)	500 (16.7 USD/day)
No. of stalk cut (stalk/household)	126.67	150	100		

*Powdered sugar/Sugar flakes*					
Daily production (kilograms/day)	11.5	13	10	1,560 (52 USD/day)	1,200 (40 USD/day)
No. of stalk cut (stalk/household)	150	150	150		

*Vinegar*					
Daily production (liters/day)	38.21	200	7.5	1,200–2,000 (40–66.7 USD/day)	99.98 (3.3 USD/day)
No. of stalk cut (stalk/household)	130	400	20		

*Thatch*					
Daily production (rows/day)	60	100	40	4,000 (133.3 USD/day)	160 (5.3 USD/day)

1 USD = 30 THB.

**Table 2:** The level of nipa palm product problems.

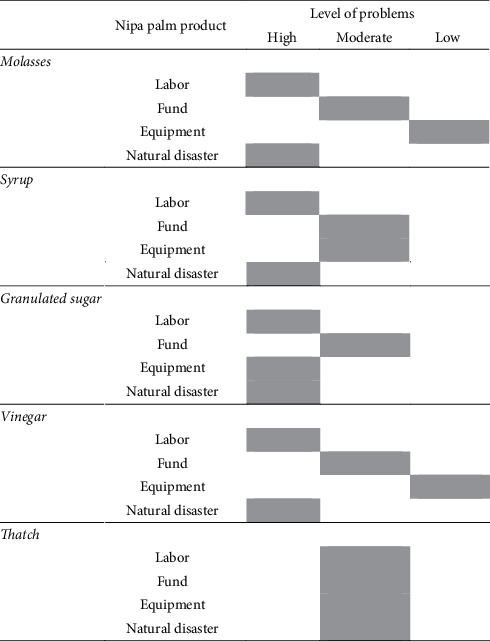

## Data Availability

The data used to support the findings of this study are included within the article.

## Supplementary Materials

(Supplementary Materials) Supplementary material 1: the map of Pak Phanang district and global positioning system (GPS) and geographic information system (GIS) to specify the geographic location of local products. Supplementary material 2: the structured interview that was used to collect the information on the utility of nipa palm product information.
