# Supplementary material for: Sustainable Nipa Palm (Nypa fruticans Wurmb.) Product Utilization in Thailand
Source: Scientifica (Cairo). 2020 Sep 25;2020:3856203. doi: 10.1155/2020/3856203 (PMC7533023; doi:10.1155/2020/3856203)

**Supplementary Materials**

Supplementary 1: the map of Pak Phanang district, Global Positioning System (GPS), and Geographic Information System (GIS) to specify the geographic location of local products.


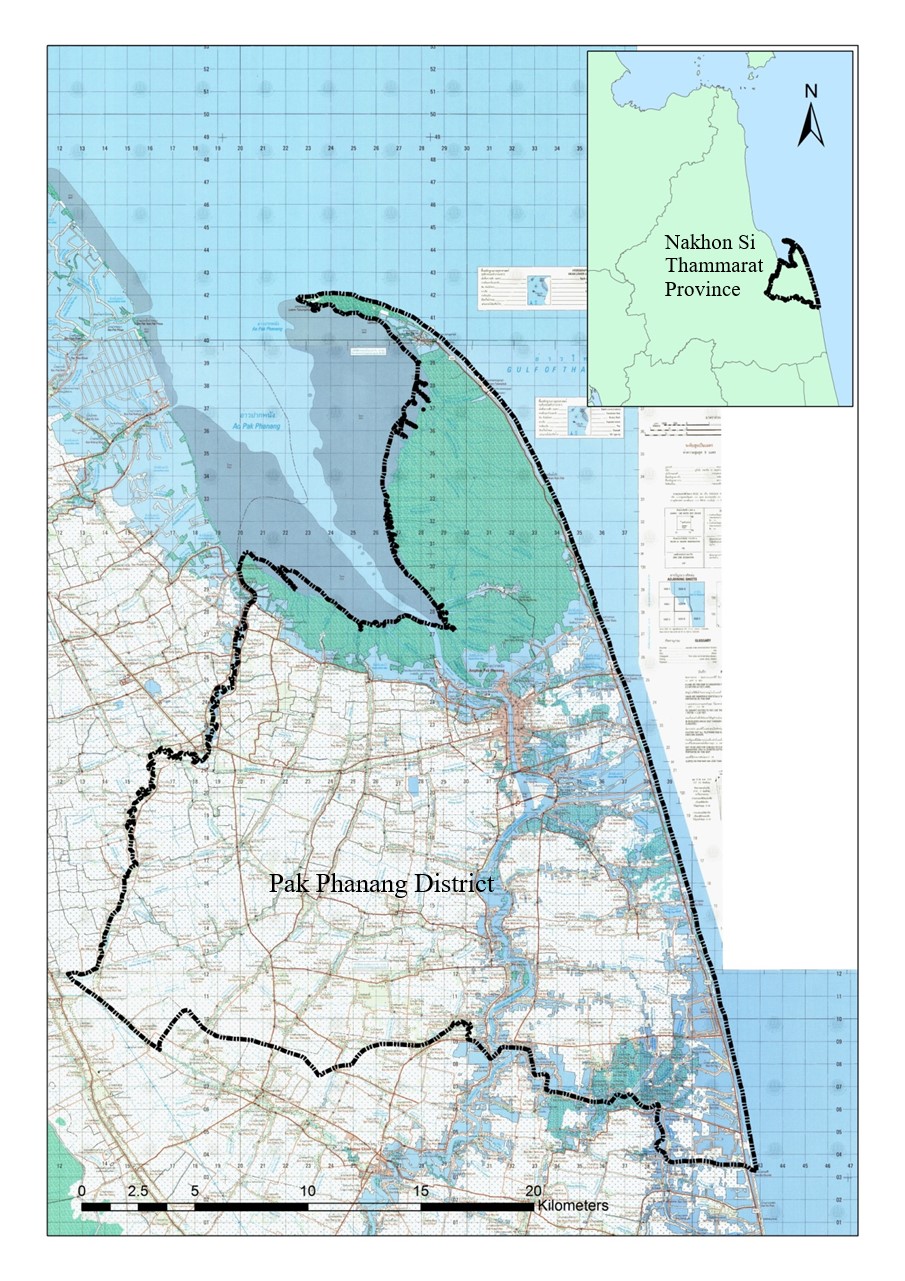


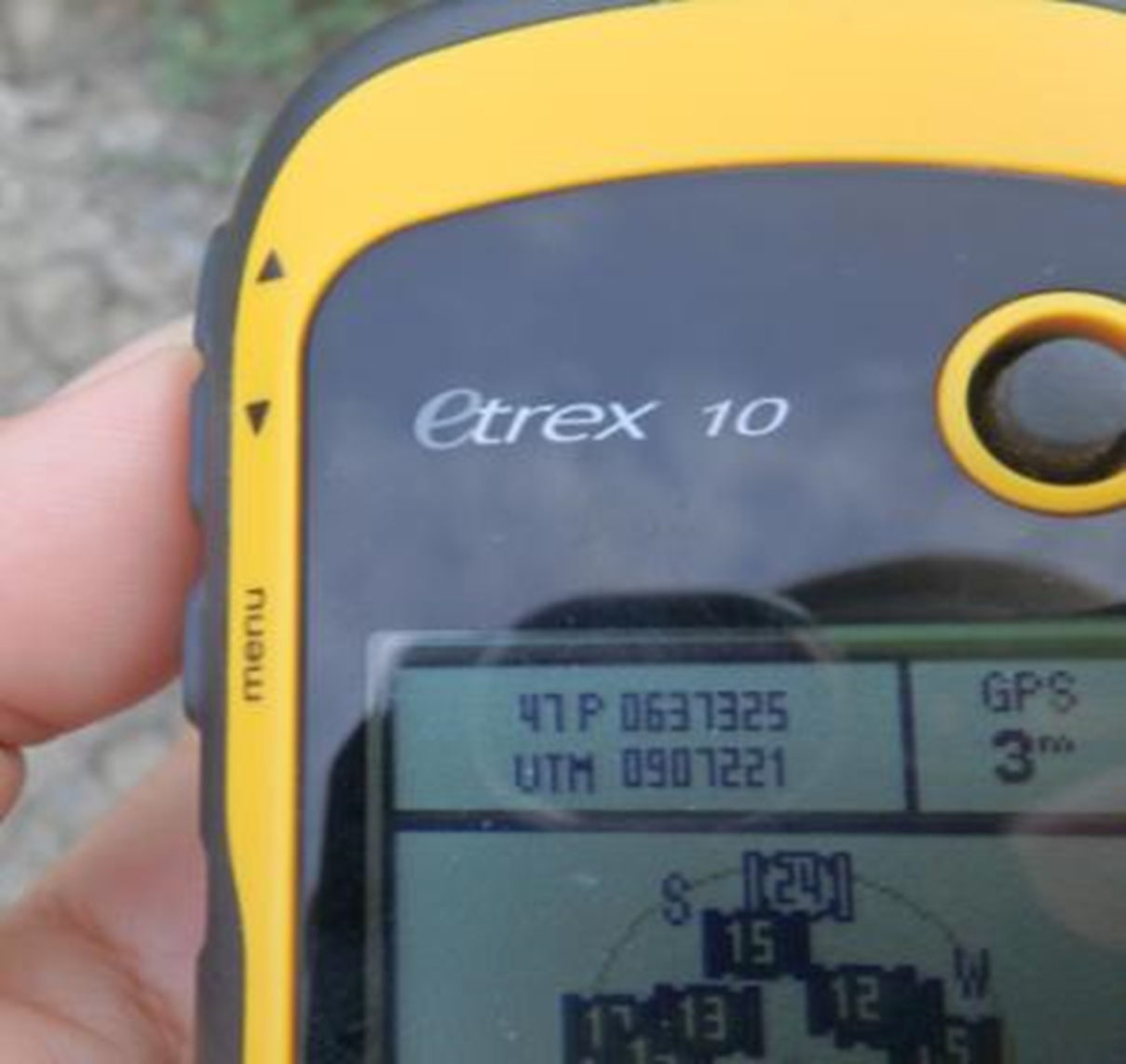


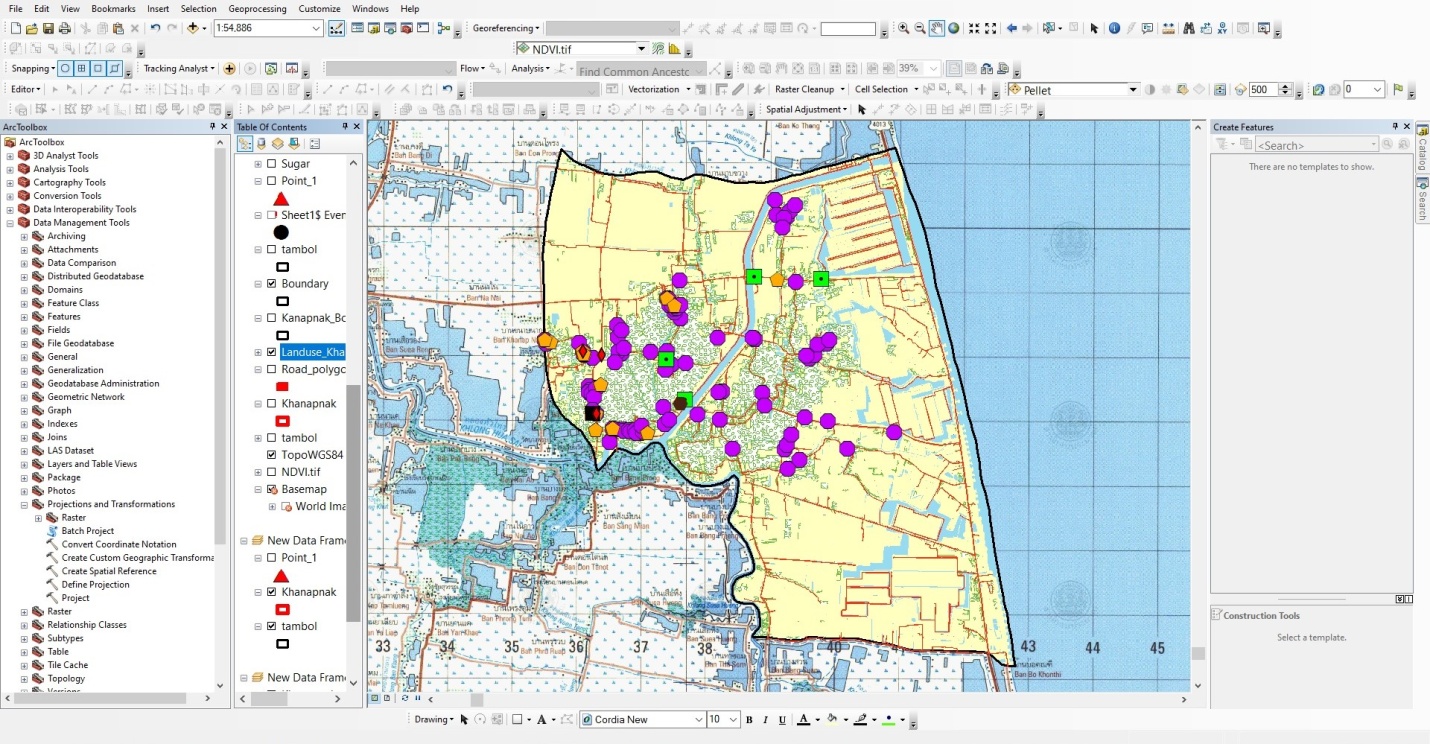


Supplementary 2: the structured interview which was used to collect the information on the utility of nipa palm product information.


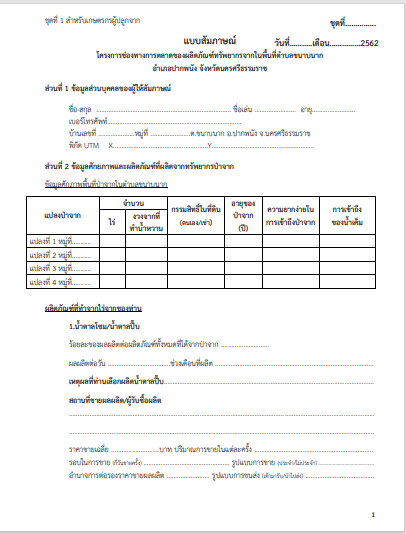


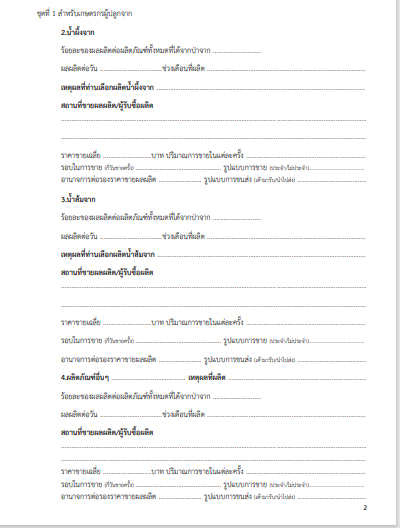


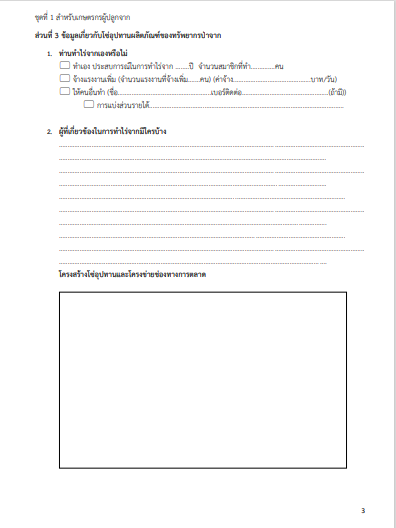


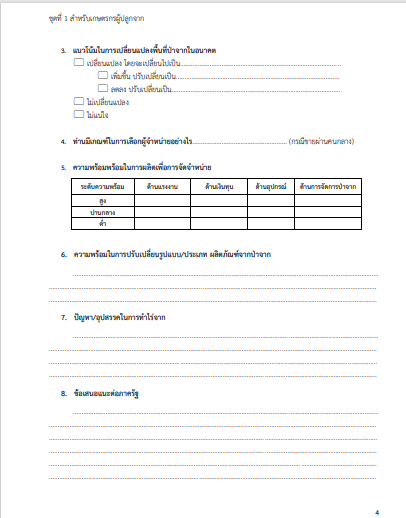

Supplement: (Supplementary Materials) — Supplementary material 1: the map of Pak Phanang district and global positioning system (GPS) and geographic information system (GIS) to specify the geographic location of local products. Supplementary material 2: the structured interview that was used to collect the information on the utility of nipa palm product information. [file 3856203.f1.docx]
